# Annotation of the Complete Genome Sequences of Bacteriophages Sara and Birdfeeder

**DOI:** 10.1128/mra.00780-22

**Published:** 2022-09-19

**Authors:** Benjamin M. Adams, Joseph B. Adams, Reganne L. Brewster, Michael S. Cutler, Anthony E. Davis, Abby H. Gallegos, Jeremy S. Hernandez, Luke H. May, Emma G. Montoya, Andrew T. Reagan, Jack F. Shurley, Anna S. Grinath, Michael A. Thomas

**Affiliations:** a Department of Biological Sciences, Idaho State University, Pocatello, Idaho, USA; Queens College CUNY

## Abstract

Sara is a siphovirus with a linear 17,362bp genome containing 25 genes. Birdfeeder is a podovirus with a circularly permuted 53,897bp genome containing 52 genes. Sara and Birdfeeder were isolated from environmental samples in Plattsburgh, NY, USA and Forest Hill, MD, USA, respectively, using Microbacterium foliorum NRRL B-24224.

## ANNOUNCEMENT

Characterizing bacteriophages improves understanding of the most plentiful biological constructs on earth and their human health applications (1, 2). As Microbacterium have been associated with plant drought resistance and meat spoilage, phages infecting this genus are of interest for the agricultural and food industries (3). Here, we describe two bacteriophages, Sara and Birdfeeder, that infect soil bacterium M. foliorum.

Phage isolation followed standard procedures (4). Sara was isolated from soil collected near the Saranac River in Plattsburg, New York, (Global Positioning System [GPS] coordinates 44.69° N, 73.46° W), whereas Birdfeeder was isolated from soil collected underneath a bird feeder in Forest Hill, Maryland, (GPS coordinates 39.559167° N, 76.423611° W). Soil samples were washed with peptone/yeast/calcium (PYCa) liquid media and bacteriophages extracted through a 0.22-μm filter. Filtrate was mixed with soft agar containing M. foliorum NRRL B-24224, overlaid on PYCa agar, and incubated at 20°C for 48 h. Sara produced small, clear pinpoint plaques while Birdfeeder produced large, haloed plaques. Both phages were purified with at least three rounds of plating.

DNA was extracted from high-titer lysates using the Promega Wizard DNA cleanup kit and prepared for sequencing using the NEBNext UltraII FS kit. DNA was sequenced using Illumina MiSeq (v3 reagents), generating 375,319 and 131,281 150-bp unpaired reads for Sara and Birdfeeder, respectively. Raw reads were trimmed and assembled using Newbler v2.9 with default parameters, yielding a single contig for each phage genome; Consed v29 used used to check genomes for completeness and accuracy and to determine phage termini (5, 6). Genome characteristics are provided in Table 1. Based on gene content similarity of 35% of higher to phages in the Actinobacteriophage database, Sara and Birdfeeder are assigned to phage clusters EE and EK, respectively (7, 8). The GC content of the genomes are consistent with other members of their respective subclusters; additionally, the GC content for Sara is similar to that of the host bacteria, M. foliorum (68.7%) (3).

**TABLE 1:** Genome characteristics for sara and birdfeeder genomes

Phage	Subcluster	Avg sequence coverage (x)	Genome size	Genome terminus arrangement	GC content	No. of protein coding genes
Sara	EE	3084	17,362bp	Linear, with 3’ single-stranded overhangs (5′-CCCGCCCCA-3′)	68.5%	25
Birdfeeder	EK	349	53,897bp	Circular	60.0%	52

Phage genomes were auto-annotated using DNAmaster v5.23.6 (http://cobamide2.bio.pitt.edu) embedded with GeneMark v2.5 (9) and GLIMMER v3.02 (10), with start sites then refined using Phage Evidence Collection and Annotation Network (PECAAN) (http://discover.kbrinsgd.org), Phamerator v454 (11) and Starterator v1.0.1 (https://seaphages.org/software). A total of 52 and 25 protein-coding genes were identified in Birdfeeder and Sara, respectively (Table 1). Using Aragorn v1.2.41 (12) and tRNA-SE v2.0 (13), no tRNAs were detected in either phage genome. Putative gene functions were determined using BLAST v2.11.0 (14) and HHPred v2.0 (15). All software used default parameters.

For Sara, structure and assembly genes span the first two-thirds of the genome. With the exception of three genes (SEA_SARA_20 to SEA_SARA_22) encoding DNA-binding proteins, all genes for Sara are transcribed rightwards. In contrast, the first third of the Birdfeeder genome encodes for several DNA metabolism genes are transcribed leftwards, followed by all rightwards-transcribed genes that include structure and assembly genes and a gene that is notably 13,479 bp-long. No immunity repressor or integrase functions could be identified for either phage. For Sara, this is consistent with its clear plaque morphology and with phages in cluster EE consisting of lytic siphoviruses. The lifecycle of phages in cluster EK is unknown.

### Data availability.

GenBank Accession and Sequence Read Archive (SRA) numbers are ON260812 & SRX14485116, respectively (Sara) and ON456346 & SRX14989441, respectively (Birdfeeder).
